# X-linked dystonia-parkinsonism: over and above a repeat disorder

**DOI:** 10.1515/medgen-2021-2105

**Published:** 2022-01-12

**Authors:** Jelena Pozojevic, Joseph Neos Cruz, Ana Westenberger

**Affiliations:** Institute of Neurogenetics, University of Lübeck, Lübeck, Germany; Institute of Human Genetics, University of Lübeck, Lübeck, Germany; Disease Molecular Biology and Epigenetics Laboratory, University of the Philippines Diliman, Quezon City, Philippines

**Keywords:** X-linked dystonia-parkinsonism (XDP), retrotransposon insertion, repeat-length polymorphism, age-related penetrance, genetic modifiers

## Abstract

X-linked dystonia-parkinsonism (XDP) is an adult-onset neurodegenerative movement disorder, caused by a founder retrotransposon insertion in an intron of the *TAF1* gene. This insertion contains a polymorphic hexanucleotide repeat (CCCTCT)_n_, the length of which inversely correlates with the age at disease onset (AAO) and other clinical parameters, aligning XDP with repeat expansion disorders. Nevertheless, many other pathogenic mechanisms are conceivably at play in XDP, indicating that in contrast to other repeat disorders, the (CCCTCT)_n_ repeat may not be the actual (or only) disease cause. Here, we summarize and discuss genetic and molecular aspects of XDP, highlighting the role of the hexanucleotide repeat in age-related disease penetrance and expressivity.

## Introduction

X-linked dystonia-parkinsonism (XDP, DYT/PARK-*TAF1*, OMIM #314250), the term first coined by Lee et al., in 1991 [1], refers to an adult-onset, severe, and frequently lethal neurodegenerative movement disorder found exclusively in individuals of Filipino origin. Although recognized several decades ago as a clearly monogenic disease, the true genetic cause of XDP has been confirmed only recently. Furthermore, the discovery of a polymorphic hexanucleotide repeat that acts as an important genetic modifier of age-related penetrance and disease expressivity has added XDP to the continuously growing list of repeat expansion disorders. This and other relevant molecular features of XDP will be discussed in the present review.

## Clinical characteristics and genetic basis of X-linked dystonia-parkinsonism

XDP usually presents in the fourth decade of life as a focal dystonia that generalizes within 5 years [2]. Generalized dystonia severely incapacitates patients and frequently leads to premature death from aspiration pneumonia and starvation. In patients surviving this disease stage, parkinsonism sets in, overlaps with the dystonia, and subsequently predominates. Of note, a small proportion of patients may develop parkinsonism as the initial symptom [1], [3], [4], [5]. Neuroimaging and postmortem brain studies define XDP as a disorder of the basal ganglia, marked by a progressive loss of medium spiny neurons in the striatum and pathological iron accumulation in the anteromedial putamen [6], [7].

**Figure 1:**
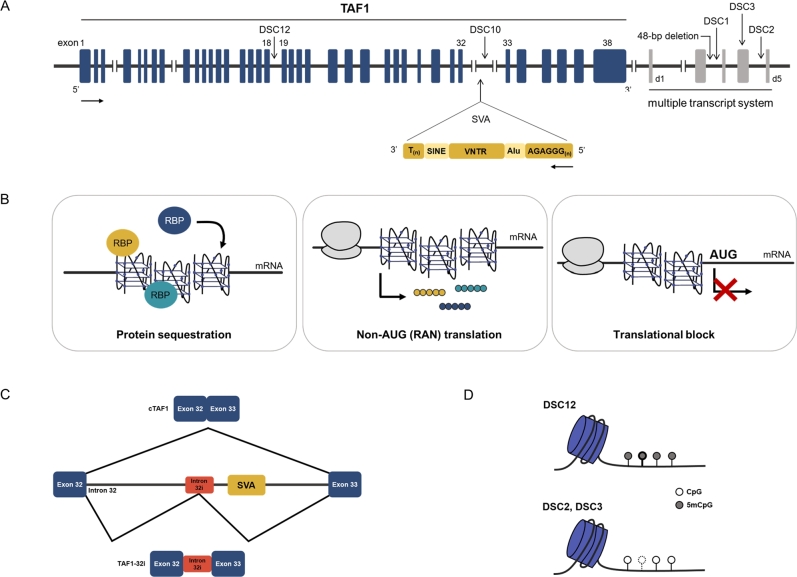
Genetic and molecular aspects of XDP. (A) The XDP haplotype within the *TAF1* gene region includes 5 DSCs, a 48-bp deletion, and an SVA retrotransposon inserted antisense. (B) The hexameric (AGAGGG)_n_ repeat within the SVA is proposed to form secondary structures, G-quadruplexes (G4s), which might act by various mechanisms, including protein sequestration, RAN translation, and/or translational block. (C) Altered splicing in XDP produces an aberrant *TAF1* transcript that contains a part of intron 32 proximal to the SVA. (D) Disease-specific single-nucleotide changes (DSCs) introduce or abolish CpG sites and, consequently, sites of DNA methylation.

XDP is endemic to the Philippines and most patients can trace their ancestry to a Philippine island (Panay), where the prevalence of this condition is 5.74 per 100,000 individuals [2]. This is explicable by the genetic founder effect, i. e., a founder mutation occurring in the ancestor common for all XDP patients. Thus, the genetic etiology of XDP is expected to be homogenous. X-linked recessive inheritance of XDP and thus the location of the putative genetic cause on the X chromosome has been formally demonstrated over 30 years ago [3]. Indeed, all XDP patients share a haplotype within a <300-kb-long region on the X chromosome, which consists of seven variants: five $\underline{d}\text{isease}$-specific $\underline{s}\text{ingle}$-nucleotide $\underline{c}\text{hanges}$ (DSC1, DSC2, DSC3, DSC10, and DSC12), one 48-bp deletion, and one ∼2.6-kb $\underline{S}\text{INE-}\underline{V}\text{NTR-}\underline{A}\text{lu}$ (SVA) element retrotransposon insertion (Fig. 1A) [8], [9]. Importantly, all seven changes within the disease-specific haplotype (“XDP haplotype”) are in linkage disequilibrium, and none of them were found in over 450 ethnically matched controls [10]. Furthermore, almost all of these variants are found either within deep intronic regions of the *TAF1* gene or in intergenic DNA segments [8], [9], [10]. The only alteration found in a nonconventional exon (DSC3), located within the “multiple transcript system” adjacent to *TAF1*, is likely untranslated [8], [11].

Given the location of the XDP-specific changes, dysfunction of the *TAF1* gene has been postulated to underlie XDP pathogenesis. *TAF1* encodes the transcription initiation factor TATA-binding protein (TBP)-associated factor 1 (TAF1), a subunit of the TFIID complex that mediates transcription by RNA polymerase II, functioning as an important regulator of the expression of numerous genes [12]. Indeed, a consistent downregulation of all *TAF1* transcripts was reported in various tissues and cell lines (e. g., blood, striatum, fibroblasts, and neural stem cells) of XDP patients compared to healthy controls [9], [13], [14]. In addition, *TAF1* was the only gene in the disease-linked region that showed a change in expression in patients in comparison to unaffected individuals without the XDP haplotype [9], [13], [14]. Subsequently, two independent studies demonstrated that the insertion of the SVA retrotransposon causes decreased *TAF1* expression, since its excision via CRISPR/Cas9-based genome editing from XDP model cells restored *TAF1* mRNA levels comparable to those seen in healthy control cells [15], [16]. Thus, out of the seven variants in the XDP haplotype, the SVA insertion is currently considered to be the most likely genetic etiology of XDP.

## The role of the (CCCTCT)_n_ repeat in XDP

Given that the causing variant is situated on the X chromosome and that XDP is inherited in an X-linked recessive manner, the majority of patients are male. Furthermore, most of them live on a genetically and geographically isolated island (Panay). Considering this genetic and environmental homogeneity, all the more surprising is the variability in AAO (20–67 years), types and distributions of the (initial) signs, and the severity of the clinical presentation that genetically confirmed XDP patients display, indicating that strong-effect modifiers of age-related penetrance and disease expressivity are likely at play in XDP. In 2017, the first genetic factor influencing the AAO in XDP was unraveled. Specifically, the length of a polymorphic hexanucleotide (CCCTCT)_n_ repeat, an inherent part of the XDP-related SVA retrotransposon, was shown to be significantly inversely correlated with AAO in a cohort of 140 XDP patients [17]. This initial report also indicated that the hexameric repeat length accounted for ∼50 % of the AAO variance among XDP patients [17]. More recently, in a cohort of 420 *TAF1* SVA insertion carriers, we found that *n* ranges from 30 to 55 repeats in blood-derived DNA samples and that in a subset of XDP patients, the repeat number correlates not only with AAO but with disease severity and cognitive dysfunction as well [5]. The repeat number showed a correlation with AAO, where for each additional increase in (CCCTCT)_n_ copy number, the AAO is decreased by 1.4 years. No association between the site of dystonia onset and repeat number was detected when comparing the craniocervical region, upper limb(s), lower limb(s), and trunk. However, in patients with first symptoms affecting the craniocervical region (eyes [blepharospasm], mouth/tongue, or neck/shoulder), the repeat number was significantly lower in those manifesting with mouth/tongue dystonia in comparison to individuals with blepharospasm [5]. Importantly, the relationship between the repeat number and initial disease manifestation (i. e., dystonia vs. parkinsonism first) was found to be indirect and dependent on AAO, meaning that the repeat number influences the AAO, which in turn determines the initial clinical manifestation [5]. The hexameric repeat number was found to be unstable within families, undergoing both expansions and contractions [5], [17]. In accordance with the existence of the parent-of-origin effect reported for other repeat disorders, the probability of the number of (CCCTCT)_n_ repeats increasing in the next generation was found to be higher when inherited from the mother. In addition, higher repeat numbers were more unstable than lower ones [5]. A very recent genome-wide association study suggested that the repeat instability in XDP at least partially results from genetic modifiers related to the MSH3 and PMS2 proteins involved in the DNA mismatch repair mechanism [18]. Namely, single nucleotide polymorphism (SNPs) in genes encoding those proteins were found to account for 13.0 % of the overall AAO variance in XDP patients, with the protective alleles delaying disease onset by 7 years [18]. In an attempt to repair partially unwound stretches of repeats, the DNA mismatch repair process likely introduces the repeat instability seen in XDP. Interestingly, a variable number of repeats was observed among different brain regions, and the repeat number was increased in brain tissue in comparison to the blood of the same individual, pointing to a tissue- or even brain region-specific effect [19]. Finally, expression analyses in blood-derived RNA of XDP patients showed that increased repeat number correlates significantly with decreased *TAF1* expression [5].

Given that earlier studies converge on decreased *TAF1* expression caused by the SVA insertion [15], [16], it is compelling to conclude that the repeats are responsible for this effect. However, the exact molecular mechanism(s) by which the hexameric repeat affects *TAF1* levels and disease expressivity remain(s) unclear. A proposed pathway involves the formation of G-quadruplexes (G4s) (the hexamer sequence on the antisense strand is AGAGGG) based on *in silico* prediction of multiple SVA domains capable of assembling into these structures, with the hexameric repeat region calculated to have the greatest G4 potential [17]. G4s are secondary RNA structures that can regulate both DNA and RNA metabolism, affecting processes such as transcription, recombination, mRNA processing, transport, and translation. The potential disease-eliciting functions of G4s have been reported to include aberrant RNA-binding protein sequestration into RNA foci, repeat-associated non-ATG (RAN) translation, and mRNA translational blockade (Fig. 1B) (reviewed in [20]). However, these various mechanisms are not mutually exclusive and neurodegeneration cannot be observed as a consequence of a single toxic mechanism [21].

## Other molecular aspects of XDP

Besides the finding that it affects *TAF1* expression in cell lines derived from XDP patients [15], [16], the SVA has also been reported to cause splicing defects, producing an aberrant *TAF1* transcript that contains a part of intron 32 of *TAF1* proximal to its insertion [15] (Fig. 1C). This intron retention transcript is rare and can be found primarily in dividing cells. Namely, a significant difference in amounts of this transcript between XDP and control cells was observed in fibroblasts, induced pluripotent stem cells (iPSCs), and neural stem cells (NSCs). On the other hand, induced cortical neurons, GABAergic neurons, and NCS-derived neurons showed only very low levels of this aberrant transcript [15]. Another recent study reported that the intron retention transcript can be detected in blood as well, and can be used as a disease-specific biomarker [22]. In accordance with the previously reported finding that this transcript ends 716 base pairs (bp) 5′ to the SVA [15], quantitative PCR expression of the *TAF1* 3′/5′ ratio (i. e., *TAF1* expression levels measured with primers targeting the end of the gene versus those measured with primers targeting its beginning) showed lower values in XDP-derived RNA samples compared to healthy controls [22].

Given that all XDP patients share the same haplotype and that this haplotype includes three single-nucleotide changes that introduce (DSC12) or abolish (DSC2, DSC3) CpG nucleotides and, consequently, sites of DNA methylation, epigenetic mechanisms seem to be another important aspect to consider in XDP (Fig. 1D). Indeed, XDP patients showed striking differences in DNA methylation at the three investigated CpG sites, compared to controls [23]. Furthermore, the XDP-specific sequence change DSC3 showed a significantly different effect on promoter activity *in vitro* in comparison to the wild-type sequence, suggesting altered transcription factor binding and a sequence-specific effect. Immunoprecipitation and mass spectrometry revealed that the DSC3 and DSC2 regions bind proteins involved in splicing and DNA- and RNA binding [23]. Even though these mechanisms still need to be investigated in XDP, it is well known that changes in DNA methylation (reviewed in [24]), as well as SNPs [25], [26], could modulate the binding of transcription factors to DNA, leading to various diseases, including Parkinson’s disease or frontotemporal dementia.

Another very recent study supports the relevance of epigenetic events in XDP. Namely, significantly decreased levels of acetylated histone H3 in exon 32 of *TAF1* were found in fibroblasts from XDP patients in comparison to control family members [27]. However, excision of the SVA in XDP-derived NSCs increased acetylated histone H3 association with *TAF1* exon 32 in comparison to unedited cells. Given that this histone modification is commonly seen at actively transcribed regions, the aforementioned observations might be related to the overall decrease in *TAF1* expression [27].

## The importance of TAF1 for the physiology of the brain

When considering the change in TAF1 expression/function as at least one of the contributors to the pathogenic mechanisms leading to XDP, an intriguing but yet unresolved question of how the alteration of a ubiquitously expressed transcription factor can affect the nervous system preferentially immediately comes to mind, especially when taking into account that missense *TAF1* variants cause a severe neurodevelopmental disorder – X-linked syndromic intellectual developmental disorder-33 (MRXS33) – characterized by delayed psychomotor development, intellectual disability, and other neurological manifestations [28]. TAF1 is an essential protein that regulates neurodevelopmental processes and its complete absence during embryogenesis is incompatible with viability in animal models [29], [30]. Postnatal CRISPR/Cas9 removal of *Taf1* in rat pups caused defects in their neonatal motor functions and altered the morphology and function of the cerebellum and cerebral cortex [30].

With respect to XDP, the existence of a neuron-specific *TAF1* transcript that would contain a neuron-specific microexon annotated as 34’ has been proposed. Decreased incorporation of this exon into the neuron-specific *TAF1* transcript could be elicited by the SVA insertion and thus contribute to XDP pathogenesis [9]. Nevertheless, a very recent study disproved this hypothesis by establishing that presence of the SVA does not influence the inclusion of microexon 34’ and that *TAF1* mRNAs containing this exon are detected at similar levels in the brains of XDP patients and controls [31].

Another theory involved a multiple transcript system (MTS) distal to the *TAF1* gene and in the region of five DSCs [8]. The MTS can be transcribed independently or spliced to *TAF1*, and one change (DSC3) is postulated to introduce a mutation into MTS transcripts [8]. In profiling experiments performed in overexpression models, transcripts that harbor DSC3 have been shown to affect the transcription of genes involved in vesicular transport in the brain and dopamine metabolism [11]. Hence, although the SVA insertion seems to influence *TAF1* mRNA levels and contribute to XDP, other variants forming the XDP haplotype may also have a role in XDP pathogenesis.

## Future research directions

Although first described 45 years ago [32], recently, XDP (re)surfaced to garner the attention of the research community, as illustrated by over 40 % of XDP-related studies having been published within the last 5 years alone. Apart from the obvious role of technological advancements, the impetus behind this surge of interest and remarkable progress in XDP research is an active group of clinicians and researchers in the Philippines whose local and international efforts assembled the momentous body of information and biospecimens. Despite being a very rare disease, the importance of XDP and its features shared with much more common neurological conditions (e. g., Parkinson’s disease and other forms of basal ganglia disorders) is being increasingly recognized. In addition, through the discovery of the disease-modifying hexanucleotide repeat, XDP became a rightful member of the dynamic repeat expansion disorder family. Intriguingly, however, the complexity of XDP reaches even further, given that it is not clear whether (and if so, to what extent) the hexanucleotide repeats are actually disease causing or are only responsible for modifying the disorder elicited by the SVA insertion itself. This question is just one in the plethora of XDP intricacies that should be addressed in future studies. Molecular mechanisms of hexanucleotide repeat action that are potentially novel and specific for XDP or shared with other repeat expansion disorders remain to be elucidated. Furthermore, the extent and relevance of the repeat number mosaicism in the brain should be investigated as well as the mechanisms causing this repeat instability. Notably, very likely, there are additional players such as genetic and epigenetic factors acting downstream or upstream from the repeats and fine-tuning their effect on the phenotype. Thus, functional follow-up studies investigating RAN translation, RNA focus formation, aberrant nucleus–cytosol transport, DNA–protein interactions, or epigenetic changes [33], [34], [35] in patient-derived biomaterial are warranted. Importantly, the fact that the number of hexanucleotide repeats and the instability modifiers jointly account for only ∼65 % of the AAO variability in the XDP patient population [17], [18] implies the existence of additional repeat-related or unrelated disease modifiers in XDP.

In any event, improved understanding of pathogenic and disease-modifying processes in XDP has important translational potential as it may aid prioritization of patients for clinical trials and indicate pertinent and plausible therapeutic approaches for XDP patients or even presymptomatic carriers. In patients in the early stages of Huntington’s disease, another repeat expansion disorder displaying striatal pathology, administration of antisense oligonucleotides resulted in dose-dependent reductions in concentrations of pathogenic mutant protein [36], while targeting G4 secondary structure was successfully applied to ameliorate the pathology of extended *C9orf72* hexanucleotide repeats, related to amyotrophic lateral sclerosis/frontotemporal dementia [37].

## Concluding remarks

XDP is a rare but clinically and genetically multifaceted disorder. The recent years have seen remarkable progress in understanding the disease pathogenesis and, importantly, modification, which has opened many novel research avenues. While XDP can be observed through the prism of a repeat disorder, it is also a disease linked to the non-coding genome, structural variants and single nucleotide alterations, aberrant splicing, epigenetic status, and transcriptional changes and regulation. Whether these are different sides of the same Rubik’s cube or multiple independent entities each acting in its own way to concomitantly contribute to XDP pathogenesis remains to be elucidated.
